# Adsorption of a Helical Filament Subject to Thermal Fluctuations

**DOI:** 10.3390/polym12010192

**Published:** 2020-01-10

**Authors:** M.-K. Chae, Y. Kim, A. Johner, N.-K. Lee

**Affiliations:** 1 Department of Physics and Astronomy, Sejong University, Seoul 05006, Korea; 2 Institute Charles Sadron, CNRS 23 Rue du Loess, 67034 Strasbourg CEDEX 2, France

**Keywords:** semiflexible polymers, polymers at interfaces, biopolymers, helical filaments, adsorption

## Abstract

We consider semiflexible chains governed by preferred curvature and twist and their flexural and twist moduli. These filaments possess a helical rather than straight three-dimensional (3D) ground state and we call them helical filaments (H-filament). Depending on the moduli, the helical shape may be smeared by thermal fluctuations. Secondary superhelical structures are expected to form on top of the specific local structure of biofilaments, as is documented for vimentin. We study confinement and adsorption of helical filaments utilizing both a combination of numerical simulations and analytical theory. We investigate overall chain shapes, transverse chain fluctuations, loop and tail distributions, and energy distributions along the chain together with the mean square average height of the monomers $\langle z^{2}\rangle$. The number fraction of adsorbed monomers serves as an order parameter for adsorption. Signatures of adsorbed helical polymers are the occurrence of 3D helical loops/tails and spiral or wavy quasi-flat shapes. None of these arise for the Worm-Like-Chain, whose straight ground state can be embedded in a plane.

## 1. Introduction

When a large macromolecule is attracted towards an adsorbing wall by a short range surface force field acting along its contour, cooperative adsorption can take place [1]. If the surface is facing a somewhat concentrated solution an adsorption layer builds up. This opens a route for easy coating and modification of the interfacial properties, adsorbed amounts of a few μM/m${}^{2}$ of monomers are often enough. Such coatings are widely used in applications [2], as steric protection of liposomes [3] and particulate drug carriers [4] or (somewhat denser) anticorrosion protection [5]. They are not so resilient against high shear stress but can often self-repair.

**Figure 1:**
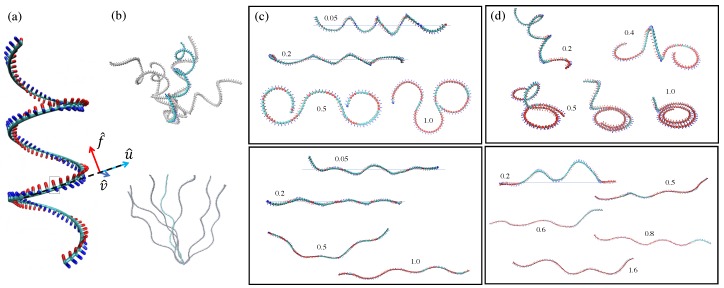
Various shapes of super-Helical filaments. (**a**) The helical shape of the ground state conformation of H-filaments ($\omega_{1}=0.81\sigma^{-1}$, $\omega_{2}=0$, and $\omega_{3}=0.10\sigma^{-1}$). The local twist and curvature are represented by a set of orthogonal vectors $\left\{\vec{u},\vec{f},\vec{v}\right\}$ along the filament backbone. $\vec{f},\vec{v}$ are defined in the material frame orthogonal to the local tangent vector $\vec{u}$. Top panels in (**b**–**d**) show H-filaments with γ>1, ($\omega_{1}=0.81\sigma^{-1}$, $\omega_{2}=0$, and $\omega_{3}=0.10\sigma^{-1}$), where circular shapes are ground states when squeezed. Bottom panels in (**b**–**d**) show H-filaments with γ<1, ($\omega_{1}=0.09\sigma^{-1}$, $\omega_{2}=0$, and $\omega_{3}=0.19\sigma^{-1}$), where wavy shapes are ground states when squeezed. The localized parts of H-filaments (|z|<0.2σ) are shown in red. (**b**) Bulk conformations subject to thermal fluctuations. (**c**) Typical conformations of H-filaments confined by harmonic potential $V\left(z\right)=\frac{1}{2}kz^{2}$ and (**d**) adsorbed under a localized surface potential $U_{ads}\left(z\right)=4\epsilon \left[{\left(\sigma /z\right)}^{12}-{\left(\sigma /z\right)}^{6}\right]$. The numbers are the strength of harmonic potential *k* and the surface potential ϵ. The figures with horizontal lines indicating the line of z=0 are side views. The flexural modulus B=50σk${}_{B}$T and twist modulus C=B/2.

In this contribution we consider the adsorption of macromolecules which have helical shape using an augmented worm-like chain model that we call helical-model (or H-model). The molecules considered here have helical radii larger than the filament diameter and are called superhelical filaments (Figure 1). This is different from double-stranded DNA (ds-DNA) in the B-form [6] and the Holmes helix of actin [7].

Indirect evidence of superhelical structure was reported in various systems. Taxol-stabilized microtubules manifest wiggly shapes and the images have been interpreted as projected helices [8]. Similar wiggly shapes are also observed from carbon nanotubes [9]. It is further reported that microtubules can convert to (open) superhelical filaments of tubulin hetero-dimers in forams. In a simulation study [10], such a behavior is reproduced and it is interpreted as a loss of longitudinal interactions between dimers. Coiled-coil structures of actin can be induced by tropomyosin [11] that itself has helical structure [12]. A superhelical filament model is introduced to describe motions of bacterial flagellar filaments [13,14]. The motion of spiral bacteria originates from helical deformation induced by a helically-wrapped actin-like fiber [15]. In these examples, helices could be easily observed because they are moderately fluctuating. It was realized that the behavior of taxoled microtubules is more complex [16,17] than just the Kratky Worm-Like-Chain (WLC) model [18], corroborating earlier observations of wiggly shapes in Carlier’s lab [8].

In various circumstances, the helical shapes are smeared out by strong thermal fluctuations. Recently, actin [19] and vimentin [20] filaments were studied under double confinement in microfluidics channels and manifest shapes unexpected for WLCs. The more precise images of vimentin have delivered data that have been successfully fitted [21] by the helical filament model. When F-actin is confined close to a wall by depleting agents, short circular actin filaments are detected with a much larger abundance than expected from the WLC model [22,23]. These findings were interpreted by the H-filament model [24]. However, no precise fit was attempted. Although there are only fragmented evidences at present, secondary (strongly) fluctuating superhelical shapes are expected to form on top of the Holmes helix for actin or of the specific local structure of vimentin. The abundance of, more or less fluctuating, superhelical structures motivates our theoretical study.

The helical filament model augments the WLC model adding a preferred twist and preferred curvature, which are both to be expected from filaments constructed by stacking repeats around their connecting axis. For very flexible filaments, helicity may only be locally relevant. The 3D ground state of this model is a helix and we call it helical or H-model. While the ground state of the WLC, which is a straight line, can be embedded into the adsorbing flat surface, the helix cannot be without costly deformation of its shape. When strictly forced into the surface, the helical filament adopts 2D conformations, which are either wavy or circular (or a mix of those) [24,25]. For a fully flexible (ideal) chain, the ground state is highly degenerate and the flat states only constitute a subset of almost vanishing measure [26,27]. The super helices considered here are subject to strong thermal fluctuations around their ground state, hence can be easily forced onto the adsorbing surface (see Figure 1). Typically, energies of the order of the thermal energy per monomer are sufficient to ensure adsorption.

Our H-filament model is purely mechanical and does not explicitly consider any chemical changes. This model [24,28,29] and other related ones [30,31] have been used previously in somewhat different studies. The strict 2D (hyper-strongly adsorbed) state of the H-model was described previously [24,28] and the induced deformation of an elastic surface analyzed [29]. Dynamics and elastic responses are discussed in [25]. Adsorption of inherently twisted tapes was studied by Quint et al. [30]. Adsorption of these superhelical twisted tapes has some relevance to side by side adhesion of twisted tapes [31] and the further assembly of polypeptide tapes in Alzheimer’s disease [32].

Adsorption is a competition between gain in interaction energy upon flattening into the surface potential and cost of confinement free energy to do so. In this contribution, we study the equilibrium characteristics of an adsorbed helical filament combining numerical simulations and analytical theory. As a preliminary to adsorption, we study characteristics of confined H-filaments together with WLCs without preferred twist and curvature but with explicit twist degrees of freedoms (WLCT). For simplicity, confinement is ensured by a harmonic potential minimal at the penetrable reference plane z=0. In Section 2, we present data obtained by MC simulation study for both confined and adsorbed H-filaments. The confined WLCT filament results are shown for comparison. Our numerical findings are interpreted theoretically using an analysis of the H-model in Section 3. Our conclusions are presented in Section 4.

## 2. Simulations

### 2.1. Model

We use the density of states (DOS) method in the scheme of Monte Carlo simulations [33,34,35,36] to study a helical chain governed by the following Hamiltonian (in discrete representation), as also used in previous work [24].

(1) $$H_{el}=\frac{1}{2}\sum_{i}\left[B{\left({\left(\Omega_{1}\right)}_{i}-\omega_{1}\right)}^{2}+B{\left({\left(\Omega_{2}\right)}_{i}-\omega_{2}\right)}^{2}+C{\left({\left(\Omega_{3}\right)}_{i}-\omega_{3}\right)}^{2}\right].$$

We model a H-filament as a helical chain consisting of N−1 links of length b≈σ. We write the hamiltonian in terms of bond vectors ${\hat{u}}_{i}$ and two additional sets of unit vectors ${\hat{v}}_{i}$ and ${\hat{f}}_{i}$. Two vectors ${\hat{v}}_{i}$ and ${\hat{f}}_{i}$ are defined in the material frame and are orthogonal to the tangent ${\hat{u}}_{i}$ of the centerline [24,30,37] (Figure 1a).

The local curvature $\Omega_{1},\Omega_{2}$ and local torsion component $\Omega_{3}$ along the chain can be obtained by ${\left(\Omega_{1}\right)}_{i}=\left({\hat{v}}_{i+1}-{\hat{v}}_{i}\right)\cdot {\hat{u}}_{i}$, ${\left(\Omega_{2}\right)}_{i}=-\left({\hat{f}}_{i+1}-{\hat{f}}_{i}\right)\cdot {\hat{u}}_{i}$, and ${\left(\Omega_{3}\right)}_{i}=\left({\hat{f}}_{i+1}-{\hat{f}}_{i}\right)\cdot {\hat{v}}_{i}$, respectively. The centerline curvature and the twist of the ground state conformation are determined by the prescribed values of $\omega_{1},\omega_{2}$, and $\omega_{3}$. The directions of the vectors $\left\{\vec{u},\vec{f},\vec{v}\right\}$ are optimized for Hamiltonian, Equation (1) (see Figure 1). Note that the prescribed curvatures and twist are the components of a vector defined in the material frame. With $\omega_{3}\neq 0$, the filament has intrinsic twist so that the direction of $\hat{v}$ and $\hat{f}$ do not necessarily match with the directions of normal and binormal vectors. Fluctuations around the helical ground state are governed by the bending and twist moduli, *B* and *C*. Additionally, the monomer–monomer interactions are modeled by the fully repulsive Lennard–Jones (LJ) potential: $U_{LJ}\left(r\right)=4\epsilon \left[{\left(\sigma /r\right)}^{12}-{\left(\sigma /r\right)}^{6}+1/4\right]$ for $r<2^{1/6}\sigma$ and 0 elsewhere.

We choose parameters $l_{p}=B/k_{B}T=50\sigma$, $l_{t}=C/k_{B}T=25\sigma$ so that the lengths of considered filaments (∼90σ) are comparable with their persistence lengths (see Figure 1a,b). The 3D ground state of the hamiltonian $H_{el}$ (Equation (1)) is a helix satisfying the preferred curvature and twist everywhere. Setting $\omega_{2}$ =0, which makes $\omega_{1}$ the preferred curvature ω, $\omega_{3}$ being the preferred twist τ, the parameters of the helix are: helical radius $R=\frac{\omega }{\omega^{2}+\tau^{2}}$, helical pitch $H=\frac{2\pi \tau }{\omega^{2}+\tau^{2}}$, and helical period along the chain $\lambda_{H}=2\pi /\sqrt{\omega^{2}+\tau^{2}}$ [38]. When squeezed, the chain shape becomes (locally) circular if all twist is expelled (twist free state). Depending on the parameters, (nearly) twist free regions are separated by twist-kinks where a twist of π is localized and where the shape has an inflection point (see Figure 1). The elastic energy for a single twist-kink inserted in an infinite circular shape reads $E_{k}=\pi \left(\sqrt{\gamma }-1\right)l_{t}k_{B}T\tau$. Here, $\gamma =\frac{4}{\pi^{2}}\frac{B\omega^{2}}{C\tau^{2}}$ measures the ratio between the bending energy cost and twist energy cost [24]. For the values B=2C considered in the simulations, $\gamma =\frac{8}{\pi^{2}}\frac{\omega_{1}^{2}}{\omega_{3}^{2}}$. In the general case [39], the twist-kink energy depends on boundary conditions. The extension of the twist-kink is characterized by the length $\lambda_{t.k}=\omega^{-1}\sqrt{\frac{C}{B}}$ [24].

Below, we consider two representative cases of H-filaments: (i) $E_{k}>0\left(\gamma >1\right)$; and (ii) $E_{k}<0\left(\gamma <1\right)$. (i) We set $\omega_{1}=0.18\sigma^{-1}$, $\omega_{2}=0\sigma^{-1}$, and $\omega_{3}=0.10\sigma^{-1}$; then, γ=2.6 and the twist-kink has the elastic energy cost $E_{k}=4.9k_{B}T$. Squeezed 2D ground state shapes are circular with no twist-kinks, for ideal chains without excluded volume. (ii) We set parameters $\omega_{1}=0.09\sigma^{-1}$, $\omega_{2}=0\sigma^{-1}$, and $\omega_{3}=0.19\sigma^{-1}$; then, γ=0.18. The isolated twist-kink energy is estimated to be negative ($E_{k}=-8.6k_{B}T$) and the squeezed ground state is wavy containing several twist-kinks. For H-filaments, the (average) number of twist-kinks and the (free) energy of the chain are then fixed by the repulsion between twist-kinks and the constraints. The 3D ground state helices for both parameter sets have the same helical radius of R=4.78σ, close helical periods along the chain $\lambda_{H}\approx 30\sigma$ and different helical pitches, P=15.0σ and P=27.0σ. In the simulation, we take a chain of N=90 monomers (about three helical periods), throughout. If $\omega_{1}=\omega_{2}=\omega_{3}=0$, the ground state shape recovers a straight line as in the simple WLC model but taking into account twist degrees of freedom. We also consider such filaments in comparison with helical filament.

To gain insight into adsorption/confinement of a helical polymer chain at thermodynamic equilibrium, we explore the phase space of conformations. We estimated the density of state (DOS) using a flat histogram MC scheme first introduced by Wang and Landau [33,34,35,36]. From DOS, the free energies and the equilibrium conformational averages can be obtained after Boltzmann weighting according to the Hamiltonian. The simulation method is similar to that described in [24] and it is described in Appendix A.

In Figure 1, we show typical shapes of confined/adsorbed H-filaments for γ>1 and γ<1. The confinement of the H-filament is ensured by the harmonic potential $V\left(z\right)=\frac{1}{2}kz^{2}$, where the distance *z* measures the distance from the desired confinement plane [25]. To study adsorption of H-filament, the surface is represented by an array of Lennard–Jones (LJ) beads of diameter *b* similar to monomer beads and the bead-wall interactions were modeled by the localized LJ potential well: $U_{ads}\left(z\right)=4\epsilon \left[{\left(\sigma /z\right)}^{12}-{\left(\sigma /z\right)}^{6}\right]$. Here, ϵ and σ represent the strength and range of the surface potential, respectively. Below, ϵ is expressed in thermal units $k_{B}T$ and lengths are measured in units of σ.

### 2.2. Simulation Results: H-Filaments Confined by a Harmonic Potential

**Figure 2:**
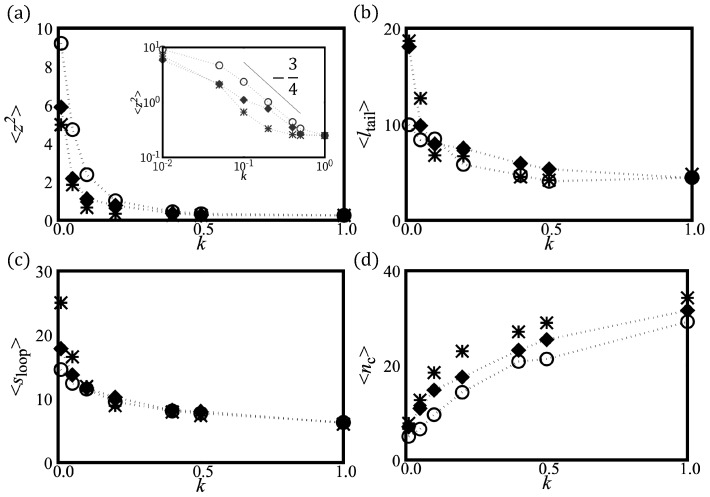
Various measured properties of H-filaments, consisting of N = 90 monomers, confined by a harmonic potential $V\left(z\right)=\frac{1}{2}kz^{2}$. One end is anchored at z=0. Symbols ∘, filled ⋄, and *, respectively, stand for: (i) γ>1; (ii) γ<1; and WLCT ($\omega_{1}=\omega_{2}=\omega_{3}=0$). (**a**) The height fluctuation $\langle z^{2}\rangle$ as the function of the stiffness *k* of the harmonic potential. The solid line represents analytical calculation in weak fluctuation limit (Equation (11)) for Case (i), $\omega_{1}=0.18\sigma^{-1}$, and $\omega_{3}=0.10\sigma^{-1}$. The inset shows mean squared height $\langle z^{2}\rangle$ as a function *k* on log-log scale. The solid line is used to guide the eye for the exponent in relation $\langle z^{2}\rangle \propto k^{-3/4}$ expected for the WLC. (**b**) Average tail length $\langle l_{\mathrm{tail}}\rangle$; (**c**) average loop length $\langle s_{\mathrm{loop}}\rangle$; and (**d**) mean number of confined monomers $\langle n_{c}\rangle$.

We first investigate shapes of H-filaments which are under the influence of the harmonic potential $V\left(z\right)=\frac{1}{2}kz^{2}$. We measured average squared height $\langle z^{2}\rangle$ and loop and tail distributions for various strength *k* of the harmonic potential, measured in units of $k_{B}T/\sigma^{3}$ (see Figure 2). The average value of $\langle z^{2}\rangle$ becomes chain length independent for a filament localized in the harmonic potential. As *k* increases, $\langle z^{2}\rangle$ decreases monotonically. For k>0.2, we found that the chain is strongly localized near z=0 and $\langle z^{2}\rangle$ is of order of unity (see Figure 2a) for γ>1. Such localization is achieved at weaker confinement potential for γ<1. The typical chain conformations are shown in Figure 1 for: (i) γ>1; and (ii) γ<1. For strong localization, the WLC behavior $\langle z^{2}\rangle \propto z^{-3/4}$ is recovered in Cases ((i) and (ii)). The strongly confined shapes with γ>1 contain two (very) localized twist-kinks and are almost circular elsewhere, while shapes with γ<1 are wavy with several twist-kinks. We also show side views of chains for k≤0.2; chain fluctuations in height decrease with increasing *k*. For strong potential (k>0.2), the 3D helical structure deforms and is lying flat closer to the surface. The flat conformation with twist-kinks is preferred over the conformations with overlap keeping preferred curvature.

**Figure 3:**
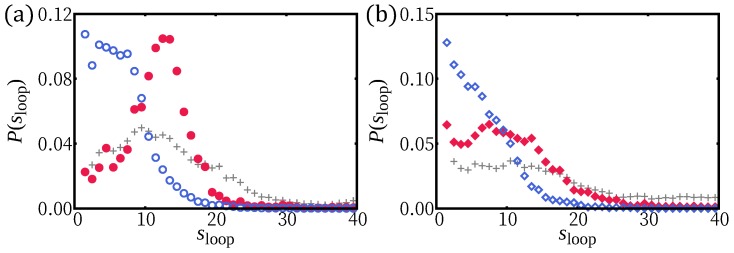
The loop length distributions for: (**a**) γ>1 (∘); and (**b**) γ<1 (⋄). Loop length is defined as the segment length that consecutively belongs to |z|>0.2. Two representative regimes, weak confinement regime *k* = 0.05 and strong confinement regime k=1.0, are shown by filled and empty symbols, respectively. The loop distribution with k=0 is shown as gray symbols (+) for comparison. For γ>1, the loop length making half of the 3D helical period is most prevalent at weak confinement. Smaller loops are equally common for γ<1, where the H-filament is more confined (see Figure 1a). Strongly confined segments show almost flat distributions for γ>1 and two sub-populations for γ<1.

We counted the number of strictly confined monomers ($n_{c}$) of which the center position lies in the range of −0.2<z<0.2. This population is increasing with *k* (Figure 2c). Small sections of chain are slightly lifted (shown as blue in Figure 1) away from the confinement plane wherever twist kinks are located. The average size of height fluctuation is nonetheless weak $\langle \delta z^{2}\rangle ∼1$. Despite that the chain is lying almost flat, the number of small lifted section reflects the number of helical periods and the number of curvature flips. Below, we define loops as strands outside the potential minimum (|z|>0.2). Both averages of loop lengths and tail lengths monotonically decrease with increasing *k* (see Figure 2b,d). In Figure 3, we show loop length distributions for γ>1 and γ<1. For γ>1, the loop length distribution has a peak at length ∼15 at weak confinement and this peak corresponds to half the helical period $\lambda_{H}$. At weak confinement, contours clearly go out of plane and return to the surface and the shapes are reminiscent of three-dimensional helical structures. They build a peak at a loop length of about half the helical period. Such a peak disappears in the distribution in the strong confinement regime and height fluctuation of strongly confined segments show flat distributions up to the loop length 10 (see Figure 3a).

For γ<1, at weak confinement, smaller loops can be equally common because H-filaments can be flattened by inserting twist-kinks. For strong confinement, the loop distribution has a shoulder suggesting two sub-populations. One sub-population originates from the circular parts and is similar to the distribution for γ>1, while the extra small loop population originates from twist-kinks and is cut at $\lambda_{t.k}\approx 7\sigma$. A similar small loop sub-population is expected in Case (i) but the cut-off $\lambda_{t.k}\approx 3\sigma$ is affected by the discreteness. (One could speculate whether the peak at very small loops is related to this effect.)

### 2.3. Simulation Results: H-Filaments Adsorbed in a Localized Surface Potential

Below, we study adsorption of H-filaments in the localized potential well $U_{ads}$.

#### 2.3.1. Adsorption of H-Filaments, γ>1

**Figure 4:**
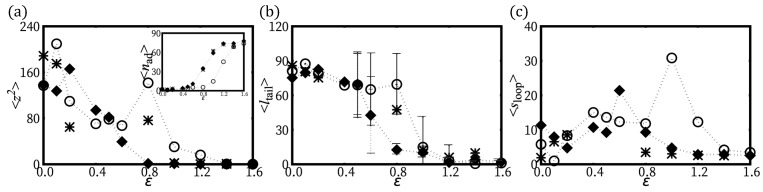
H-filaments adsorbed in a localized surface potential $U_{ads}$. Various properties are measured for a H-filament of length S=90σ with one of its ends anchored at z=0. Symbols ∘ and filled ⋄ stand for the case of γ>1 and γ<1, respectively. WLCT case at strong/weak adsorption regimes are represented as *. (**a**) $\langle z^{2}\rangle$ at various strengths of surface potential ϵ. The inset shows the average number of the adsorbed monomers (|z|<0.2). (**b**) Average tail length $\langle l_{\mathrm{tail}}\rangle$; and (**c**) average loop length $\langle s_{\mathrm{loop}}\rangle$ at various ϵ.

In Figure 4, we summarize several physical quantities representing the adsorption behavior of H-filaments with γ>1 due to the localized surface potential. At weak adsorption, the whole shape remains 3D helix. The mean squared height $\langle z^{2}\rangle$ in general decreases as ϵ increases but there is a regime 0.2<ϵ<0.6 where $\langle z^{2}\rangle$ shows a more complex variation with ϵ. This corresponds to the change of shape documented in Figure 1c, with the formation of a 3D loop followed by a 3D tail. A 3D loop forms, as both ends are localized flat and the middle part (almost) retains its 3D helical shape (Figure 1c). For increasing ϵ, the nucleated spiral shapes at both ends become enlarged. For larger ϵ(>0.6), we find that shapes with only one spiral and a 3D tail become stabilized, as shown in Figure 1c. (Remember the boundary conditions are asymmetric with one end being attached to the surface.) The average length of $\langle l_{\mathrm{tail}}\rangle$ monotonically decreases as ϵ increases and has large fluctuation ∼30σ close to the adsorption transition. At strong adsorption ϵ>1.0, the average tail length reaches to a few monomer lengths (Figure 4b). In the adsorbed regime, loops are rare and the poor statistics translates in noisy data in $\langle s_{\mathrm{loop}}\rangle$ (Figure 4c).

**Figure 5:**
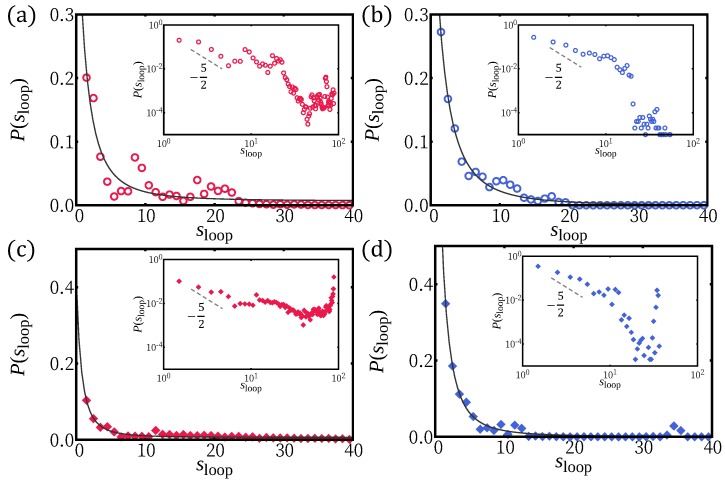
The loop length distributions of H-filament γ>1 (**a**,**b**) and γ<1 (**c**,**d**) for the two representative values of ϵ: weak adsorption regime (ϵ = 0.1) (**a**,**c**); and strong adsorption regime (ϵ = 1.0) (**b**,**d**). The envelop of distributions in general follows WLC statistics $s^{-5/2}$ (with an offset ≲1 in *s*), which is represented as solid line.

Small loops are expected to be ruled by bending energy with a length distribution $\propto s^{-5/2}$, following WLC statistics [40]. The loop length distribution shows this trend for all adsorption strength simulated; nonetheless, a wide power law regime does not develop. There are local maxima at quantized loop lengths for small ϵ, reflecting the 3D helical structure (Figure 5a,b).

**Figure 6:**
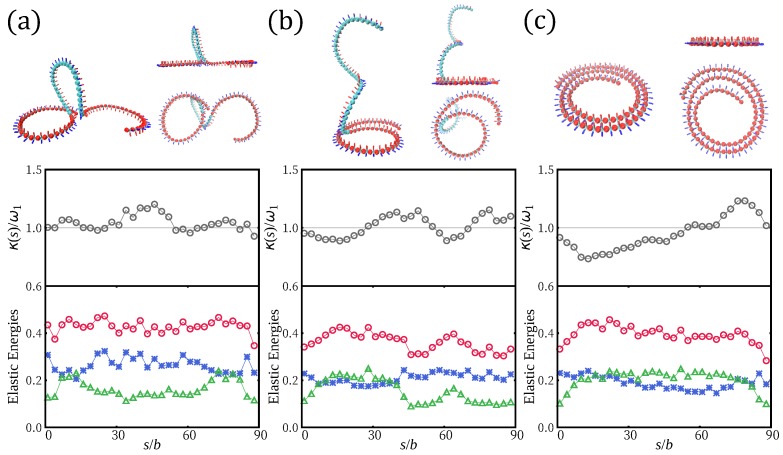
Three representative shapes of adsorbed H-filaments (γ>1) at: (**a**) ϵ = 0.4; (**b**) ϵ = 0.8; and (**c**) ϵ =1.6. The localized parts are shown in red. The middle panels show measured local curvatures κ(s) relative to the preferred curvature $\omega_{1}=0.181$. Local curvatures $\kappa \left(s\right)=\sqrt{\Omega_{1}^{2}+\Omega_{2}^{2}}$ (∘) are averaged over several similar conformations along the contour *s*. Lower panels show twist energy density (green ▵), bending energy density (blue *), and the sum of these two (red ∘).

For large ϵ, the height of the chain is mainly determined by the tail. We analyze the typical shapes of the adsorbed H-filament in the transition regime and in the tail dominated regime. In Figure 6, we plot local curvatures κ(s) and mechanical energy densities (twist energy density $\epsilon_{tw}$ and bending energy density $\epsilon_{b}$) along the contour length *s* for some typical shapes of the adsorbed helix at ϵ=0.4,0.8, and 1.6. We take the averages over several similar configurations. As twist is frustrated upon adsorption, the average twist energy density of 3D loops and 3D tails is relatively smaller than that of adsorbed parts. The local curvature can be better optimized for the adsorbed section. However, the curvature is fluctuating around the preferred value of $\omega_{1}$. The averaged local curvature κ(s) converges to $\omega_{1}$ everywhere along the chain (shown as gray circle in middle panels of Figure 6). If we define a turn, for a flat configuration, such that the tangent recovers its orientation, the average curvature along the turn is inversely proportional to its length. As shown in Figure 1c, there are bulges with a localization depending on the configuration. For a specific adsorbed flat spiral section (Figure 6c), the curvature of the inner circle is slightly larger than the optimal curvature $\omega_{1}$ and the local curvature of the outer circle is smaller than $\omega_{1}$. The shape at ϵ=0.4 includes 3D loop in the middle section (Figure 6a), where twist energy is low. In shapes at intermediate adsorption strength, both tail and spiral can coexist (Figure 6b). The 3D tail part clearly has lower twist energy. The adsorption of the tail section would go on as long as the curvature energy away from the optimal value is less than the excess adsorption energy with respect to the adsorption threshold for the ideal filament (without excluded volume). While the tail part (monomer index > 40) keeping 3D helical structure has lower mechanical energy, the elastic energies of adsorbed spiral are slightly larger; these costs are compensated by the energy gain upon adsorption.

#### 2.3.2. Adsorption of H-Filaments, γ<1

We also investigated the adsorption behavior for H-filaments with γ<1. Typical conformations of a chain are shown in Figure 1d at various values of surface potential ϵ and several physical quantities are shown in Figure 4. Similar to Case (i) (γ>1), the average squared height $\langle z^{2}\rangle$ decreases with increasing ϵ and the adsorption transition where $\langle z^{2}\rangle ∼1$ occurs at smaller value of ϵ∼0.8. Around ϵ=0.8, the number of adsorbed monomers $\langle n_{\mathrm{ad}}\rangle$ has its steepest increase. The free energies $F(\bar{z^{2}})$ have minimum at $\bar{z^{2}}∼0$ for ϵ>1 (not shown). We also obtained the average tail length $\langle l_{\mathrm{tail}}\rangle$ and the average loop (strand lying |z|>0.2) length $\langle s_{\mathrm{loop}}\rangle$ (Figure 4b,c). In the weak adsorption regime, the average loop length is ∼7σ, markedly smaller than $\lambda_{H}/2∼15\sigma$, but the average tail length $\langle l_{\mathrm{tail}}\rangle ∼70-80\sigma$, is comparable to $\langle l_{\mathrm{tail}}\rangle$ of Case (i) (γ>1). For larger ϵ, the adsorbed part adopts a flat wavy shape and the remaining builds a 3D tail. The tail length $\langle l_{\mathrm{tail}}\rangle$ drops to ∼10σ at adsorption transition ϵ∼0.8. The 3D loops are less common than for Case (i), i.e., γ>1, because they can more easily flatten into a twist-kink. The loop length distributions are shown for ϵ=0.1 and ϵ=1.0 (Figure 5c,d). At strong adsorption, the loop length distribution reflects height fluctuations of adsorbed segments, which is reminiscent of the WLC (Figure 5d).

## 3. Theory

We consider a helical chain, which is characterized by four parameters: two geometrical parameters, the preferred curvature $\omega_{1}$ and twist $\omega_{3}$, and two mechanical parameters, the bending modulus B and twist modulus C. The elastic energy of a helical WLC can be written as a function of the local curvatures $\Omega_{1,2}$ and twist $\Omega_{3}$ as:

(2) $$E=\frac{1}{2}\int_{0}^{S}B\left[{\left(\Omega_{1}-\omega_{1}\right)}^{2}+\Omega_{2}^{2}\right]+C{\left(\Omega_{3}-\omega_{3}\right)}^{2}ds.$$

The 3D ground state is a helix satisfying the preferred curvature and twist everywhere, as discussed in the model section.

To proceed and assess the shape of H-filaments, it is convenient to express the $\Omega_{i}$ through the Euler angles $\Omega_{1}=\phi^{\prime }\sin\theta \sin\psi +\theta^{\prime }\cos\psi ,$$\Omega_{2}=\phi^{\prime }\sin\theta \cos\psi -\theta^{\prime }\sin\psi$, and $\Omega_{3}=\phi^{\prime }\cos\theta +\psi^{\prime }$, where (.)${}^{\prime }$ designates the derivative with respect to the arc length parameter *s* along the chain. The twist $\Omega_{3}$ is the sum of the centerline torsion and the intrinsic twist $\psi^{\prime }$.

We constrain the chain to a plane by imposing θ=π/2. The 2D ground state satisfies the Euler–Lagrange equations:

(3) $$\begin{array}{c} \phi^{\prime }=\omega_{1}\sin\psi . \end{array}$$

(4) $$\begin{array}{c} \psi^{\prime \prime }+\frac{\omega_{1}^{2}}{2c}\sin2\psi =0. \end{array}$$

The Euler–Lagrange equation admits wavy and circular solutions, the actual ground state being determined by the parameters of the system and the boundary conditions (external torque). The circular solution satisfies the curvature $\omega_{1}$ everywhere and frustrates the torsion $\omega_{3}$.

In the following, we consider a (large) 2D circular portion which must be unstable without a proper confining constraint. Another case of interest would be an infinite 2D H-filament with a single curvature inversion along the filament.

### 3.1. Instability of the 2D Configuration

We impose that the flat circular shape is maintained/imposed outside the section of interest and try to figure out which wavelengths are most unstable. We stick to a quadratic expansion of the energy in terms of the small deviations $z^{\prime }=\pi /2-\theta$, $\epsilon =\Phi -\omega_{1}s$ and η=ψ−π/2. Our main interest is in the small coordinate *z* perpendicular to the plane where the edges of the H-filament are maintained. To quadratic order:

(5) $$\begin{array}{c} E-E_{0}=\frac{1}{2}\int B\left[{\left(\epsilon^{\prime }\right)}^{2}+\omega_{1}^{2}\eta^{2}+{z^{\prime \prime }}^{2}-2\omega_{1}\eta z^{\prime \prime }\right]+C\left[{\eta^{\prime }}^{2}+\omega_{1}^{2}{z^{\prime }}^{2}-2\omega_{3}\epsilon^{\prime }z^{\prime }+2\omega_{1}\eta^{\prime }z^{\prime }-2\omega_{1}\omega_{3}z^{\prime }-2\omega_{3}\eta^{\prime }\right]ds \end{array}$$

where the energy of the reference circular arc $E_{0}=\int C\omega_{3}^{2}/2ds$ has been subtracted. It is convenient to express the energy in thermal units making the moduli equal to the persistence lengths. Further, we take the monomer size as unit length making *z* dimensionless. This units are identical to those used in the simulations presented above. To quadratic order, the out of plane fluctuation *z* couples to both the bending and torsion fluctuations, which do not couple to each other. To proceed, we introduce Fourier components of the fluctuations ϵ, *z*, and η. While ϵ and *z* only enter the energy by derivatives that have zero average along the strand under consideration, η enters directly and its average along the strand $\bar{\eta }$ is a random variable subject to the constraint that η vanishes at the boundaries. We implement this mode analysis below in this section. For the time being, we take the naive continuous Fourier transform with the change of notation $\eta \to \bar{\eta }+\eta$. The quadratic form in Equation (5) can then be recast as

(6) $$E-E_{0}=\int d\frac{q}{2\pi }H\left(q\right)$$

where *q* is the Fourier conjugate of *s*.

(7) $$\begin{array}{ccc} H(q) & = & \frac{1}{2}B\left\{q^{2}\epsilon_{q}\epsilon_{-q}+\omega_{1}^{2}\eta_{q}\eta_{-q}+q^{4}z_{q}z_{-q}+q^{2}\omega_{1}\left(\eta_{q}z_{-q}+\eta_{-q}z_{q}\right)\right\}\\ & + & \frac{1}{2}C\left\{q^{2}\eta_{q}\eta_{-q}+q^{2}\omega_{1}^{2}z_{q}z_{-q}-q^{2}\omega_{3}\left(\epsilon_{q}z_{-q}+\epsilon_{-q}z_{q}\right)+q^{2}\omega_{1}\left(\eta_{q}z_{-q}+\eta_{-q}z_{q}\right)\right\} \end{array}$$

We determined the spectrum and eigenvectors of the quadratic form in Equation (7). The high-*q* modes are always stabilized by the bending energy and all three eigenvalues are positive. Lowering *q*, the 2D shape turns unstable when the lowest eigenvalue becomes negative, which is also seen on the sign of the determinant *D* of the quadratic form.

(8) $$8D=q^{4}\left(B^{2}Cq^{4}-Cq^{2}\left(2B^{2}\omega_{1}^{2}+C^{2}\omega_{3}^{2}\right)+CB\omega_{1}^{2}\left(B\omega_{1}^{2}-C\omega_{3}^{2}\right)\right)$$

The bracket in the determinant has two real roots for all values of he parameter. Two cases may be distinguished according to the value of the parameters.

- For $B\omega_{1}^{2}<C\omega_{3}^{2}$, only the largest root $q_{1}^{⋆}$ is positive. All long wavelength $q<q_{1}^{⋆}$ are unstable. This corresponds to the case where wavy shapes are favored over circular ones in 2D.
- For $B\omega_{1}^{2}>C\omega_{3}^{2}$, the two roots $q_{1}^{⋆},q_{2}^{⋆}$ are positive. The low q-modes are stable. Intermediate wavelength $q_{2}^{⋆}<q<q_{1}^{⋆}$ are unstable. This suggests (it is only a linear stability study) that, when the helical chain goes on the surface, it does so by forming loops of intermediate length. This corresponds to the case where circular shapes are favored over wavy ones in 2D.

For the values of the parameters investigated, the unstable eigenmodes are almost pure *z*, pure ϵ, and pure η. The partition function Z in the Gaussian approximation can be traced over ϵ and ν to obtain the effective Hamiltonian $H_{z}$ for the fluctuation in *z*:

(9) $$H_{z}=\frac{1}{2}S_{0}^{-1}\left(q\right)z_{q}z_{-q}$$

with

(10) $$S_{0}^{-1}\left(q\right)=q^{2}\frac{B^{2}Cq^{4}-Cq^{2}\left(2B^{2}\omega_{1}^{2}+C^{2}\omega_{3}^{2}\right)+CB\omega_{1}^{2}\left(B\omega_{1}^{2}-C\omega_{3}^{2}\right)}{B(B\omega_{1}^{2}+Cq^{2})}.$$

**Figure 7:**
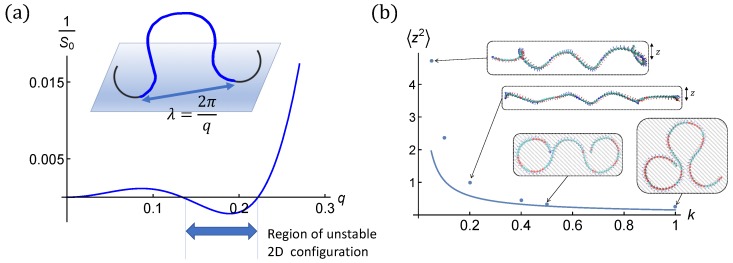
(**a**) The bare inverse structure factor $S_{0}^{-1}\left(q\right)$ defined in Equation (10) (i.e., k=0) for $\omega_{1}=0.18$ and $\omega_{3}=0.10$. Boundary conditions impose flat circular shapes outside of the section of interest. Note the region of instability indicated by negative $S_{0}^{-1}$. The harmonic potential of strength *k* would shift the inverse structure factor upwards by *k*, $S_{k}^{-1}\left(q\right)=S_{0}^{-1}+k$. Hence, k>0.005 stabilizes the flat helical chain against small amplitude fluctuations. In practice, larger *k* is required to ensure the validity of the quadratic expansion. (**b**) The height fluctuation $\langle z^{2}\rangle$ in weak fluctuation limits as a function of the stiffness f*k* of the harmonic potential for helical filament (i), $\omega_{1}=0.18\sigma^{-1}$, $\omega_{3}=0.10\sigma^{-1}$. Analytical calculation following Equation (11) is shown as solid line and the simulation data for filaments with one end anchored are shown in dots together with typical conformations at some *k*-values. Flat configurations at *k* = 0.5 and 1.0 are captured from the top view.

The effective Hamiltonian $H_{z}$ reflects the instabilities discussed above. The inverse structure factor in Equation (10) is represented in Figure 7.

### 3.2. H-Filaments Maintained by a Harmonic Surface Potential

Let us now consider the case where the H-filament is subjected to a harmonic potential $V\left(z\right)=\frac{1}{2}kz^{2}$ per unit chain length. The Hamiltonian becomes $H_{z}=\frac{1}{2}S_{k}^{-1}\left(q\right)z_{q}z_{-q}$ with $S_{k}^{-1}\left(q\right)=S_{0}^{-1}+k$. If the harmonic potential is stiff enough (if *k* overcompensates the minimum of $S_{0}^{-1}\left(q\right)$), the chain is localized against small height fluctuations. Otherwise, it fluctuates away from the surface and builds a loop reflecting the 3D helical structure.

Let us focus on the stable regime. The structure factor $S_{k}\left(q\right)$ corresponds to the height-height correlation function in the harmonic potential and to the (linear) response function to a force in the *z*-direction. It is of interest to calculate the fluctuation of *z*:

(11) $$\langle z^{2}\rangle =\int_{-\infty }^{+\infty }\frac{dq}{2\pi }S_{k}\left(q\right)$$

When the instability is approached, the Gaussian approximation fails, well before the fluctuation reaches that of the 3D helix, $R_{H}^{2}/2$. The integral in Equation (11) can be calculated; we may think about it in the complex plane in terms of residues. Under strong confinement (large k), we enforce high *q*-poles (in all other scales) and the fluctuations are controlled by the bending stiffness *B*. In this very limit the fluctuations in *z* of the H-filament are identical to those of a WLC and $\langle z^{2}\rangle =\frac{1}{2\sqrt{2}\sqrt[{4}]{Bk^{3}}}$ is proportional to $B^{-1/4}k^{-3/4}$.

In the twist expulsion limit, this regime is met provided $k\gg B\omega_{1}^{4}$, in our case $k\gg 0.05\sigma^{-3}$. This is one order of magnitude more than needed to formally stabilize fluctuations to quadratic order (Figure 7).

The weak fluctuation limit is met for k>0.2 in our case. For small fluctuations (large *k*), the fluctuations obtained by simulation match the analytical result of Equation (11). When the fluctuations become very small, the contribution of the twist-kink regions, not accounted for by the theory, can be seen (k=1). Figure 7b shows $\langle z^{2}\rangle$ in the weak fluctuation limit as given by Equation (11) together with simulation data. The agreement is good for k>0.2 as expected.

Further, for the WLC in the harmonic potential, there is a preferred loop length. Considering the lowest mode representation of a flat loop of height *h*, $z\left(s\right)=h\sin\left(\pi s/s_{\mathrm{loop}}\right)$, we get an estimate for the optimal loop length $s_{opt}\approx 3^{1/4}\pi l^{1/4}k^{-1/4}$. This suggests optimal loop lengths somewhat larger than observed in simulations. Except for the strongest confinement regime the helical chain retains some of its 3D helical structure which biases the loop length towards half the 3D helical period. The loop length is also consistent with the half-period of the less stable wavelength (Figure 7). For the strongest confinements (k=1), simulation data do not show a narrow peak in the loop distribution (Figure 3d), which is consistent with the shape of $S_{k}^{-1}\left(q\right)$ that is pretty flat (see Figure 7), with some weak structure, in the intermediate *q*-range. Here, the loop length associated with *q* is half a period. Below, we refine our approach and consider a finite H-filament strand and implement the (chosen) boundary conditions strictly.

### 3.3. Stability of a Finite Helical Strand

As all quantities ϵ, η, and *z* are assumed to vanish at both edges (s=0 and s=L) of the strand of length *L*, we may quite generally decompose them in the Fourier modes $\psi_{n}=\sqrt{\frac{2}{L}}\sin\left(\pi ns/L\right)$. Below, we use the Fourier decomposition $\epsilon =\sum_{n=1}\epsilon_{n}\psi_{n}$, $\eta =\sum_{n=1}\eta_{n}\psi_{n}$, $z=\sum_{n=1}z_{n}\psi_{n}$. We remember that the fluctuations of the fields $z_{n}$ are not independent as we also require $z^{\prime }$ to vanish at both edges. This imposes $\sum_{n=1}nz_{n}=0$ and $\sum_{n=1}{\left(-1\right)}^{n}nz_{n}=0$. As usual, there is a high frequency cut-off of the sums when the wave length approaches the finite chain thickness and we regularize physical quantities. (Note that the constraint makes a distinction between even and odd modes and does not have an obvious equivalent in the continuous representation.) The energy can then be expressed as a sum over the modes similar to Equation (7) with integrals over reciprocal space replaced by sums over modes. Following the same steps, we can trace over the fields $\epsilon_{n}$ and $\eta_{n}$ to obtain:

(12) $$\begin{array}{c} E=\sum_{n=1}\frac{1}{2}S_{n}^{-1}z_{n}^{2}\\ S_{n}^{-1}=S_{k}^{-1}\left(q=\pi n/L\right). \end{array}$$

To obtain the partition sum, we further trace over $z_{n}$ under the constraint $z^{\prime }\left(0\right)=z^{\prime }\left(L\right)=0$. This reduces the number of (independent) modes by two. The partition sum can now be obtained (see the Appendix B), for example, by tracing over the $z_{i}$, i≥3, $Z={\left(\det\left(S_{i,j}^{-1}\right)\right)}^{-1/2}$. The determinant is obtained as:

(13) $$\det\left(\frac{S_{i,j}^{-1}}{2}\right)=\prod_{i=1}\frac{S_{i}^{-1}}{2}\times \left(\sum_{i=1,odd}i^{2}S_{i}\right)\times \left(\sum_{i=2,even}i^{2}S_{i}\right).$$

The first factor corresponds to the unconstrained system, and the corresponding free energy contribution reads $F_{free}=\frac{1}{2}\sum_{i=1}\log\left(S_{i}^{-1}\right)$. Taking all contributions into account:

(14) $$L\langle z^{2}\rangle =\sum_{i=1}S_{i}-\frac{\sum_{i=1,odd}i^{2}S_{i}^{2}}{\sum_{i=1,odd}i^{2}S_{i}}-\frac{\sum_{i=2,even}i^{2}S_{i}^{2}}{\sum_{i=2,even}i^{2}S_{i}}.$$

The first term $\sum_{i=1}S_{i}$ is the discrete analog of Equation (11), with q→πi/S (note that in Equation (11) the integral over *q* extends from −∞ to +∞). The extra terms, induced by the constraints, have no continuous counter parts. We assume *k* large enough for all the $S_{i}^{-1}$ to be positive, which defines the stable region of parameters. All the series involved in Equation (14) converge and are further extensive (grow linearly with *L*) in the quasi-continuous limit. The contribution of the extra terms to $\langle z^{2}\rangle$ hence vanishes when L→∞, as it should. Let us decrease *k* in the stable regime; the first term diverges when the unstable q-region first approaches one of the discrete modes. This increase is compensated to leading order by the extra terms (being the first unstable mode even or odd). It is generally expected that adding a constraint ($z^{\prime }=0$) sharpens the transition for a finite system (see, for example, the buckling transition). This issue is marginal here and is not discussed further. In the simulation, the typical loop should experience finite size corrections (possibly with softer boundary conditions).

### 3.4. H-Filaments Adsorbed in a Localized Surface Potential

In the following, we propose a qualitative theory suited to interpret the simulation data mainly for γ>1, before briefly commenting on the case γ<1. While the Worm-Like Chain (WLC) is a useful reference, the behavior of H-filaments essentially differs by their ground state geometry, which cannot be fully imbedded in the localized surface potential of width Δ smaller than the helical radius. In contrast to the harmonic confinement, free loops entirely localized outside the surface potential can form, which retain the 3D helical structure at least qualitatively. On the other hand, as discussed above, for a strongly adsorbed chain, short wavelength out of plane fluctuations remain dominated by the flexural modulus *B*. In practice, our simulations on finite chain length invite to investigate several regimes: (i) a loosely adsorbed regime where the helical structure hardly deforms but the chain is trapped on the surface by its scarce surface contacts; (ii) an adsorbed state where sections lying almost flat inside the wall potential coexist with large 3D helical loops (H-loops); and (iii) a strongly adsorbed regime where the chain gives up its 3D structure but in a 3D helical tail (the chain is grafted by one end). The latter case corresponds, for example, to the strongly adsorbed configurations presenting spiral-type of shapes with an inner radius of curvature slightly larger than the preferred one, one turn almost at the preferred curvature, and increasingly frustrated outer turns; eventually this frustration overcomes the gain by adsorption and a finite tail is more favorable. The length of the tail decreases with the adsorption strength. Although H-loops are always expected on a long adsorbed helical polymer chain, they are constrained to connect to the surface twice, which also tends to quantize their length. The tails are less constraint.

Let us consider a 3D H-filament grafted to the impenetrable adsorbing surface by one end and free to rotate about its grafting point in the upper half-space. The adsorption potential is characterized by an extension Δ and a strength ϵ. The helical chain will give up its rotational degrees of freedom for adsorption strengths $\epsilon >\epsilon_{0}$ corresponding to an adsorption energy exceeding the thermal energy (qualitatively). Assuming no deformation of the helix, this leads to the adsorption strength $\epsilon_{0}$:

(15) $$\epsilon_{0}∼\frac{1}{2n_{p}\sqrt{2R_{3}\Delta }},$$

where we assume $n_{p}$ helical periods and a helical radius $R_{3}$. For weak adsorption, we expect that a long helical chain almost retains its local 3D structure and behaves as an equivalent WLC. This regime is barely seen in the simulation. For higher adsorption strength, the helix progressively deforms to benefit from the surface potential at the expense of elastic energy. A second adsorption threshold $\epsilon_{c}$ corresponds to complete flattening of the chain inside the potential well. For the helical chain to lie flat on the surface in an optimal circular shape, it must give up its preferred twist and pay the associated energy $E_{tw}=C\omega_{3}^{2}/2$ per unit length, to be further localized in the surface potential of width Δ the ordinary squeezing energy $E_{sq}∼\frac{1}{\Delta^{2/3}B^{1/3}}$ of a WLC is to be paid per unit length. We hence get the value $\epsilon_{c}=E_{sq}+E_{tw}$. It is estimated to be 1.3, which is consistent with the estimate from the simulations (1.2–1.4) (see Figure 4b). Let us now consider the spiral-like shape; obviously, the curvature $\Omega_{1}$ cannot be optimal everywhere along the chain. In the simulation, the central region almost satisfies the curvature $\Omega_{1}$. There is hence an additional cost for the other sections including the end section. Assuming that the radius increases by δ per turn with $\Omega_{1}\delta <1$, we qualitatively get the bending energy $E_{B}\left(n\right)=\frac{1}{2}B\Omega_{1}^{2}{\left(n\delta \Omega_{1}\right)}^{2}$ per unit length in the *n*th turn where the optimal turn is labeled 0. The *n*th turn in the spiral hence adsorbs flat provided:

(16) $$\begin{array}{c} \epsilon -\epsilon_{c}>E_{B}\left(n\right)\\ \epsilon_{c}∼C\omega_{3}^{2}/2+\frac{1}{\Delta^{2/3}B^{1/3}.} \end{array}$$

This criterion is more and more demanding as turns accumulate. Close enough to the adsorption threshold, a tail develops (only one can in the simulation) collecting the monomers not satisfying this criterion. Several physical improvements may be necessary; there is a cost for the tail/adsorbed-strand junction, which may be overcompensated by the frustration of the end of the circular section (it has to comply to the free boundary conditions).

What mainly changes in the wavy shape (γ<1) is the easy formation of twist-kinks, which cures the excluded volume problem. This is a favorable alternative to the central 3D loop for moderate to strong adsorption. Although a tail can form, it is not driven by frustration (unlike in the spiral shape) but may help accommodate free boundary conditions. The moderate tail length shrinks dramatically at strong adsorption.

## 4. Conclusions

For various confinement/adsorption strengths, filaments with two different sets of preferred curvature and twist are considered. They are chosen such that the strict 2D configurations are qualitatively different (without and with twist-kinks) [24]. The obtained conformations of H-filaments are very different from each other and we would like to discuss how much is due to finite chain length effects. While, for an ideal helical filament, flat overlapping spires with optimal curvature are allowed, these overlapping spires are not allowed if excluded volume is taken into account. We observe two types of shapes:

(a) Wavy shapes or 3D loops which avoid self-crossing of the chain, where the frustration of the configuration is mainly localized at some spots along the chain.
(b) Spiral shapes, where the frustration is smeared out and terminal sections escape into 3D tails.

Shapes (a) are observed for harmonic confinement and for adsorption at intermediate adsorption strength (3D loop). Shapes (b) are encountered for stronger adsorption where a tailed spiral forms. The localized adsorption potential does not put any extra penalty on 3D sections, which are essentially free. In contrast, the harmonic potential penalizes monomers increasingly as they move away from the surface. Shapes (b) do not seem favorable for long chains. Indeed, the amount adsorbed in the spiral saturates and more and more monomers go to the tail for longer chains. For long helical chains (longer than simulated), we expect several 3D loops to form along the chain. There are a number of metastable states. This limits the efficiency of Brownian Dynamics simulation and conventional Monte Carlo sampling.

When the ideal 2D target configuration is wavy (small γ), excluded volume effect is less relevant and the transition to quasi 2D is easier both for harmonic confinement and adsorption.

Forcing a helical polymer in strict 2D space leads to characteristic shapes either circular or wavy. These shapes appear fairly well defined even for strongly fluctuating 3D helices agitated by thermal fluctuations. In practice, we cannot expect to observe strict 2D shapes but rather confinement to the vicinity of a surface (e.g., by depletion interaction) or adsorbed states, as described here.

Besides the study of the (single) H-filament itself, it is of interest to study the formation of adsorption layers. Layers of polyamide-6,6 on graphene were simulated in a coarse-grained model using Molecular Dynamics [41]. The proximal regime of small stiff loops is difficult to characterize in that work due to the very small persistence length of ∼ 1 nm, and, as expected, signatures of hyper-helicity were not observed. Quite some efforts went into the adsorption of the simpler WLC model [42]. Stiffness favors the adsorption of long strands of a WLC flat on the surface and layers may sometimes be difficult to equilibrate during the build-up. Irreversible adsorption models are then relevant [40,43], less so for H-filaments which tend to coil out of the surface.

## Figures and Tables

**Figure A1:**
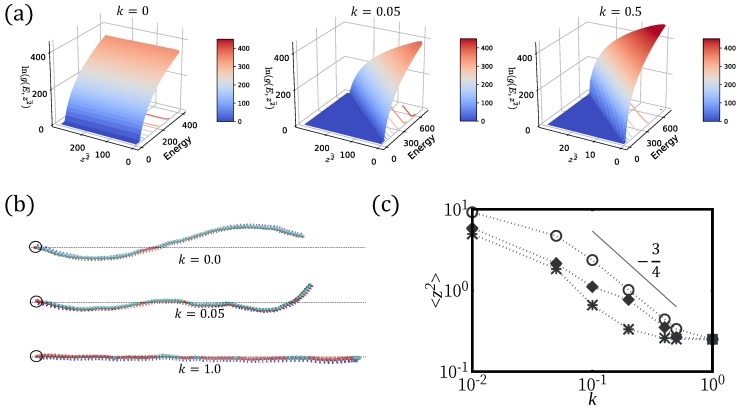
(**a**) DOS $g(E,\bar{z^{2}})$ of WLCT under various strength of harmonic potential $V\left(z\right)=\frac{1}{2}kz^{2}$, k=0,0.05 (weak confinement) and k=0.5 (strong confinement). (**b**) Loops of WLCT at various *k*. (**c**) $\langle z^{2}\rangle$ of WLCT in harmonic potential confinement in log-log scale for k=0.05,0.1,0.2,0.4, and 1.0 from top to bottom. The line is used to guide the eye and represents $\langle z^{2}\rangle \propto k^{-3/4}$.

**Figure A2:**
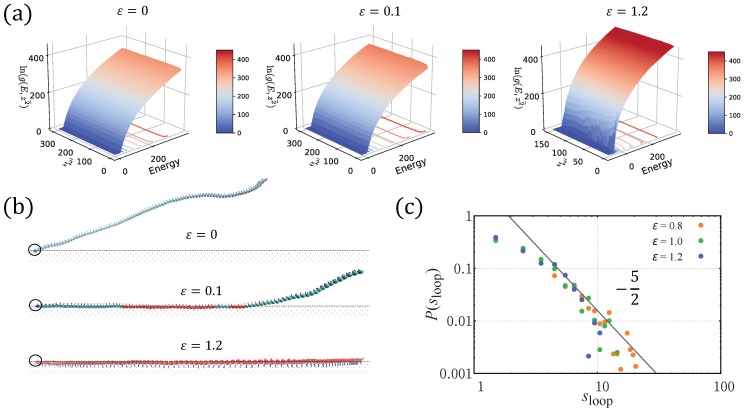
(**a**) DOS $g(E,\bar{z^{2}})$ of WLCT filament interacting with short range attractive potential. (**b**) Representative conformation of WLCT at weak (ϵ=0.1) and strong (ϵ=1.2) adsorption regime. (**c**) Loop length distribution P(s). The line represent the scale of adsorption loop length distribution $P\left(s\right)\propto s^{-5/2}$.

## Appendix A. Simulation Method

To compute an ensemble average at given squared height of $\bar{z^{2}}$, we use a flat histogram MC scheme first introduced by Wang and Landau [33,34,35,36]. We compute DOS as a two-dimensional function $g(E,\bar{z^{2}})$ sampling of two-dimensional histograms. We choose the squared extension $\bar{z^{2}}$ as a parameter, as density of states is symmetric with respect to the adsorbing plane. The new state r in phase space is accepted with a probability $p\left(r_{old}\to r_{new}\right)=min\left(1,g\left(E\left(r_{old}\right)\right)/g\left(E\left(r_{new}\right)\right)\right)$. For efficient sampling, we use the global update method introduced by Zhou et al. [44]. We apply the density of state method to H-filaments confined by harmonic potential V(z) and to H-filaments adsorbed by localized surface potential $U_{ads}$. Once $g(E,{\tilde{z}}^{2})$ is obtained, the equilibrium conformational average $\langle X\left(\bar{z^{2}}\right)\rangle$ of a quantity *X* can be obtained from the DOS after Boltzmann weighting according to the Hamiltonian,

(A1) $$\langle X\left(\bar{z^{2}}\right)\rangle =\sum_{E}g\left(E,\bar{z^{2}}\right)e^{-E/k_{B}T}X\left(E,\bar{z^{2}}\right)/Z,\;\;\;Z=\sum_{E.\bar{z^{2}}}g\left(E,\bar{z^{2}}\right)e^{-E/k_{B}T}.$$

The free energy $F({\tilde{z}}^{2})$ is given by

(A2) $$F\left({{\tilde{z}}_{0}}^{2}\right)=-k_{B}T\ln\left[\sum_{E}\sum_{{\tilde{z}}^{2}}\delta \left({\tilde{z}}^{2}-{{\tilde{z}}_{0}}^{2}\right)e^{-E/k_{B}T}\right].$$

Prior to the simulation of H-filaments, we checked that the density of states of WLCT computed using this Monte Carlo scheme faithfully reproduced the predicted results from theory. The density of states (DOS) $g(E,\bar{z^{2}})$ of WLCT with respect to the energy and mean squared height $\bar{z^{2}}$ for weak and strong confinement are shown in Figure A1. For strong confinement, the population in density of state is suppressed for large *z*.

Figure A1 represents the density of states (DOS) $g(E,\bar{z^{2}})$ of H-filament for a strength of confinement potential *k* = 0.05 and *k* = 0.5. The same method was successfully used for the strict 2D H-filament where a 2D sampling was done according to the end-to-end distance and number of twist-kinks [24]. Figure A2a shows DOS at representative strength of surface potential for weak and strong adsorption ϵ = 0.1, and 1.2. The minimum in free energy shifts to $\bar{z^{2}}\approx 0$ as the strong adsorption regime is approached. The expected scaling exponent of WLC [43] loop distributions $P\left(s_{loop}\right)\propto s_{loop}^{-5/2}$ is seen at loop length $s<l_{p}$. The calculation of DOS at a specific confinement strength takes approximately 240 h on an Intel Zenon 2.4 GHz PC running Linux. The convergence is considerably slow at the regime of adsorption transition.

## Appendix B. Derivation of Partition Sum

One may take the constraints $z^{\prime }\left(0\right)=0$, $z^{\prime }\left(S\right)=0$ into account by expressing $z_{1}$ and $z_{2}$ as sums over the other modes, although this is not the best way [45]. The idea is that all other modes are integrated out as being free and $z_{1}$ and $z_{2}$ are fixed by the constraint for each set {$z_{i}$}. The energy can then be written as a quadratic form with an effective Hamiltonian $H=\sum_{i=3,j=3}\frac{1}{2}S_{i,j}^{-1}z_{i}z_{j}$ with:

(A3) $$S_{i,j}^{-1}=\delta_{i,j}S_{i}^{-1}+ij\Delta_{i,j},$$

where

(A4) $$\Delta_{i,j}=\sigma_{i,j}\left(\frac{\sigma_{i,j}-1}{2}S_{1}^{-1}+\frac{\sigma_{i,j}+1}{2}\frac{S_{2}^{-1}}{4}\right),$$

with $\sigma_{i,j}=\frac{{\left(-1\right)}^{i}+{\left(-1\right)}^{j}}{2}$. In practice, $\Delta_{i,j}=S_{1}^{-1}$, for odd *i* and *j*; $\Delta_{i,j}=S_{2}^{-1}/4$, for even *i* and *j*; $\Delta_{i,j}=0$ otherwise. The partition sum can now be obtained by tracing over the $z_{i}$, i≥3, $Z=(\det{\left(S_{i,j}^{-1}\right)}^{-1/2}$, which is used in Equation (13) in the main text.
